# Long-Lived αMUPA Mice Show Attenuation of Cardiac Aging and Leptin-Dependent Cardioprotection

**DOI:** 10.1371/journal.pone.0144593

**Published:** 2015-12-16

**Authors:** Esther Levy, Ran Kornowski, Reut Gavrieli, Ilana Fratty, Gabriel Greenberg, Maayan Waldman, Einat Birk, Asher Shainberg, Amit Akirov, Ruth Miskin, Edith Hochhauser

**Affiliations:** 1 The Cardiac Research Laboratory, Felsenstein Medical Research Center, Tel Aviv University, Petah Tikva, Israel; 2 Cardiology Dept., Rabin Medical Center, Petah Tikva, Israel; 3 Faculty of Life Sciences, Bar-Ilan University, Ramat Gan, Israel; 4 Cardiology Dept. and Schneider Children’s Medical Center, Tel Aviv University, Petah Tikva, Israel; 5 Weizmann Institute of Science, Rehovot, Israel; Virginia Commonwealth University, UNITED STATES

## Abstract

αMUPA transgenic mice spontaneously consume less food compared with their wild type (WT) ancestors due to endogenously increased levels of the satiety hormone leptin. αMUPA mice share many benefits with mice under caloric restriction (CR) including an extended life span. To understand mechanisms linked to cardiac aging, we explored the response of αMUPA hearts to ischemic conditions at the age of 6, 18, or 24 months. Mice were subjected to myocardial infarction (MI) *in vivo* and to ischemia/reperfusion *ex vivo*. Compared to WT mice, αMUPA showed functional and histological advantages under all experimental conditions. At 24 months, none of the WT mice survived the first ischemic day while αMUPA mice demonstrated 50% survival after 7 ischemic days. Leptin, an adipokine decreasing under CR, was consistently ~60% higher in αMUPA sera at baseline. Leptin levels gradually increased in both genotypes 24h post MI but were doubled in αMUPA. Pretreatment with leptin neutralizing antibodies or with inhibitors of leptin signaling (AG-490 and Wortmannin) abrogated the αMUPA benefits. The antibodies also reduced phosphorylation of the leptin signaling components STAT3 and AKT specifically in the αMUPA myocardium. αMUPA mice did not show elevation in adiponectin, an adipokine previously implicated in CR-induced cardioprotection. WT mice treated for short-term CR exhibited cardioprotection similar to that of αMUPA, however, along with increased adiponectin at baseline. Collectively, the results demonstrate a life-long increased ischemic tolerance in αMUPA mice, indicating the attenuation of cardiac aging. αMUPA cardioprotection is mediated through endogenous leptin, suggesting a protective pathway distinct from that elicited under CR.

## Introduction

Ischemic heart disease is a major determinant of human mortality and morbidity worldwide, accounting for approximately 90% of deaths in aged Western populations [1]. Susceptibility of the heart to ischemia increases with age in man and rodents [2,3]. It is therefore likely that long-lived animal models are endowed with life-long endogenous cardioprotective mechanisms. Two such models are animals placed under caloric restriction (CR), a widely studied model in which 20–40% decrease in food intake is usually imposed [4,5], and the αMUPA transgenic mice [6].

αMUPA mice spontaneously consume less food when fed ad libitum compared to their wild type (WT) FVB/N ancestral genotype [7]. αMUPA mice exhibit a satiated and obesity-resistant phenotype primarily due to high serum levels of leptin [8,9], an adipocyte-derived hormone that regulates food intake and energy expenditure [10,11] and declines under CR [12]. These mice also show high levels of transcripts encoding an anorexigenic neuropeptide in the brain along with low levels of transcripts for several orexigenic neuropeptides [8], all known to be subjected to leptin regulation and involved in leptin-mediated satiety [13]. αMUPA mice also have low levels of the hunger-inducing hormone ghrelin, but interstingly, no change in adiponectin [8], a multifunctional adipokine inversely associated with adiposity [14,15], that increases under CR [16,17] and protects against cardiac dysfunction following myocardial infarction (MI) [18]. αMUPA mice thus exhibit a unique metabolic phenotype of low calorie intake and reduced adipose tissue yet high leptin levels but no change in adiponectin. Despite the aforementioned metabolic differences, αMUPA and CR mice share many benefits including increased life span, reduced body weight and fat mass, reduced body temperature, increased insulin sensitivity, reduced serum levels of insulin-like growth factor-1 (IGF-1) and reduced incidence of spontaneous cancers and induced tumorigenic lesions [7,8,19,20].

Numerous studies have previously demonstrated variable CR-induced beneficial changes in the heart [4,5,21,22,23,24]. In particular, long- and short-term CR accelerate the recovery of left ventricular function after ischemia/reperfusion (I/R) [25,26], and counteract the age-dependent decline in ischemia preconditioning, an endogenous protective response to brief I/R episodes [25,27]. The exact beneficial mechanisms elicited by CR in general and specifically in the heart are not completely understood. CR-induced cardioprotection after I/R was abrogated in adiponectin antisense mice [16] and after inhibiting phosphorylation of adenosine monophosphate-activated protein kinase (AMPK) [16, 28] thus implicating the adiponectin/AMPK pathway in the protective process [16,28].

The heart has not yet been studied in αMUPA mice. These mice, originally designed to express the transgenic enzyme urokinase-type plasminogen activator (uPA) specifically in the ocular lens, also show ectopic transgenic expression in the brain but not in the heart [6,20]. Here we have used these mice as a sustainable long-lived animal model by which to uncover endogenous mechanisms contributing to cardioprotection. We found that αMUPA mice demonstrate an attenuation of cardiac aging along with leptin-dependent increase in ischemic tolerance.

## Methods

### Ethics statement

**Fig 1:**
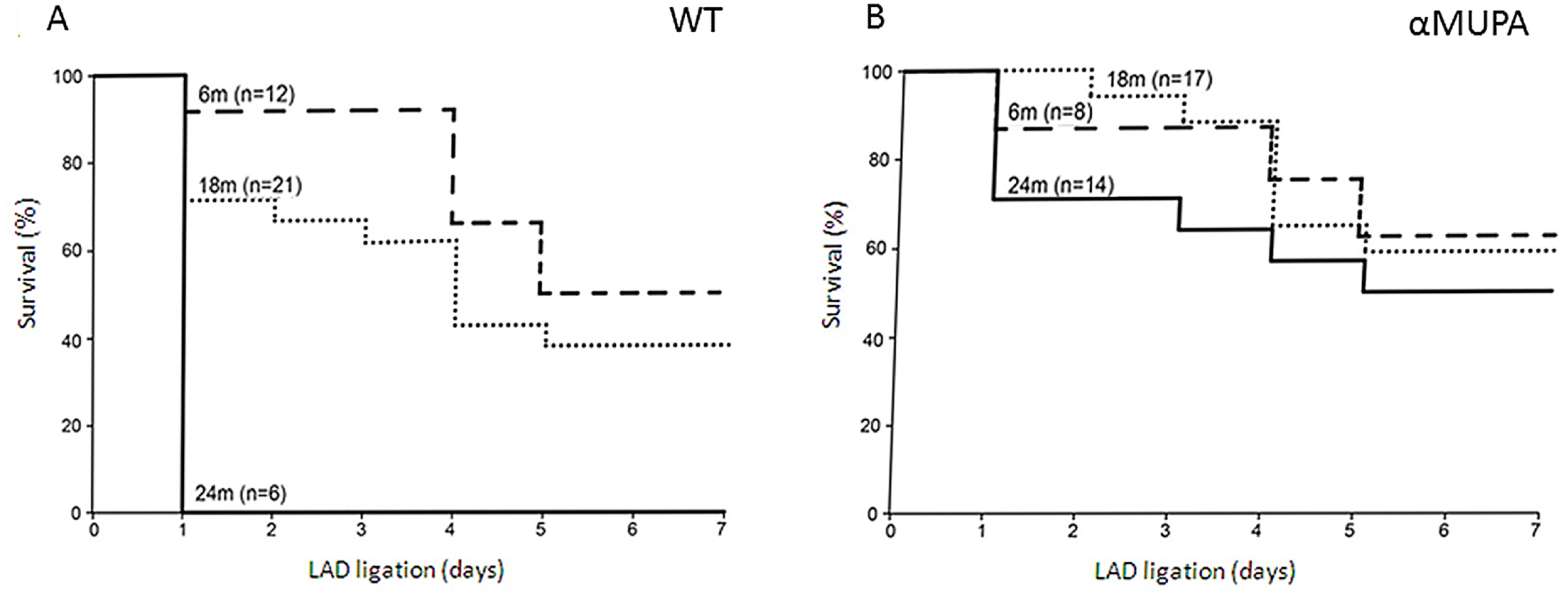
Kaplan-Meier survival curves for WT and αMUPA mice for 7 days MI. WT (A) and αMUPA (B) mice were subjected to LAD ligation *in vivo* for 7 days at the ages of 6, 18 and 24 months. The number of mice in each group is indicated in the figure. Survival was monitored after each day throughout the ischemic period. For each individual mouse, the time of death was plotted against the percent of mice still alive.

All experimental protocols, including all experimental endpoints, were approved by the Tel Aviv University Institutional Animal Care and Use Committee (IACUC) (#M-07-021 and #M-13-087), in accordance with the Guide for the Care and Use of Laboratory Animals published by the US National Institutes of Health. To induce myocardial Infarction (MI), mice were anesthetized with a mixture of ketamine/xylazine as described below. Throughout the experimental ischemic period in vivo, lasting for 24h or 7days, animals were monitored at least twice a day. All animals under MI received Aspirin (2 g/100 ml) in the drinking water to alleviate pain. When an animal appeared seriously frail, a fluid therapy was performed by subcutaneous injection of 0.2 ml saline, usually twice a day. All mice undergoing a 24h ischemic period were euthanized with 5% isoflurane inhalation. As cardiac aging is characterized by reduced ischemic tolerance, it was important to compare our long-lived mice and their WT control for this behavior. We therefore induced MI at different ages for 7 days, a period long enough to develop several important functional and histological changes described in Fig 1 and Table 1. Unfortunately, spontaneous death could have taken place as an intrinsic animal behavior under this ischemic protocol, especially in the old population, as indeed depicted in Fig 1. Any intervention trying to humanely accelerate death in this experiment could have interfered with our final conclusions. Therefore, it was of interest to avoid such an intervention in this specific case. The possibility of spontaneous mortality under prolonged ischemic stress was reviewed and approved by our ethics committee. All mice surviving the 7-day-MI period were humanely sacrificed by inhaling 5% isoflurane after undergoing ecocardiography.

**Table 1:** Cardiac functional data derived from echocardiography of WT and αMUPA mice after 7 days MI or sham operation at 6, 18 and 24 months of age.

Group	Age (months)	LVEDD (mm)	LVESD (mm)	FS (%)	HR (bpm)
WT sham, n = 9	6	2.34±0.13	1.17±0.082	50.5±1.4	526±31
αMUPA sham, n = 15	6	2.44±0.07	1.15±0.046	53.2±0.7	549±20
WT LAD, n = 9	6	2.98±0.139*	1.78±0.127 *	39.8±2 *	514±70
αMUPA LAD, n = 9	6	2.6±0.059 * ^,^ ^~^	1.42±0.056 * ^,^ ^~^	44.8±1.6 * ^,^ ^~^	538±25
WT sham, n = 23	18	2.94±0.082	1.44±0.059	50.7±0.9	523±16
αMUPA sham, n = 27	18	2.57±0.053	1.23±0.030	52.3±0.7	557±9
WT LAD, n = 16	18	3.5±0.014 *	2.2±0.010 *	38.1±1.0 *	502±18
αMUPA LAD, n = 21	18	2.9±0.095 * ^,^ ^~^	1.64±0.06 * ^,^ ^~^	43.3±1.0 * ^,^ ^~^	510±16
WT sham, n = 6	24	2.32±0.136	1.12±0.080	51.2±1.3	412±20
αMUPA sham, n = 9	24	2.42±0.088	1.18±0.040	51.8±1.0	490±23
WT LAD, n = 9	24	All mice died in less than 24 hours post LAD ligation			
αMUPA LAD, n = 9	24	3.26±0.202*	1.91±0.204*	41.3±1.4*	452±29

Data are means ±S.E.M.

*P< 0.05, pre MI vs. post MI in the same genotype at the same age.

^~^ *P*< 0.05, WT vs. αMUPA at the same age.

### Animals and experimental protocols

Transgenic αMUPA [6] and their control WT FVB/N mice, originally obtained from Ruth Miskin, Weizmann Institute of Science (Rehovot, Israel), were propagated at the homozygous state at the Felsenstein Medical Research Center-Tel Aviv University (Petah Tikva, Israel). Mice were housed in a temperature-controlled room with 12:12 h light-dark cycles with free access to standard chow (Harlan, Teklad Global Diet-18% protein) and drinking water. Female mice, unsynchronized for the estrus cycle, were used in all experiments. For CR treatment, a separate group of 8-week-old FVB/N mice were singly housed in cages and fed *ad libitum* (AL) for 2 weeks for adaptation and determination of spontaneous food intake. The average daily food intake was measured and considered 100% while calculating the CR diet. The mice were then randomly assigned into CR- and AL-fed groups. The AL group was fed AL for the rest of the experimental period. The CR group was fed weekly 95%, 85%, 75% and 65% of the AL food intake. Experiments were conducted immediately thereafter. After the 5-week CR period, the CR-fed group showed a significantly reduced body weight compared to the AL-fed group (15.6±2.3g vs. 22.4±2g, p<0.05, respectively).

### I/R in the isolated heart

The I/R procedure was conducted as we have previously described [29]. Briefly, hearts were quickly removed from heparinized (500 U/kg, i.p) anesthetized mice (5% isoflurane inhalation) and perfused with oxygenated Krebs Henseleit solution, stabilized for 20 min and subjected to 30 min ischemia followed by 20 min reperfusion. Left ventricular pressure (LVP) was determined throughout the procedure using a fluid-filled latex balloon connected to a pressure transducer, that was inserted via the left atrium into the left ventricle. LVP was recorded using the CODAS data acquisition system. Coronary flow samples were collected every 10 min. Infarct size was determined using TTC staining as we have previously reported [29].

### MI *in vivo*

Mice were treated for MI at the age of 6, 18 or 24 months, as indicated. Ischemia was achieved by left anterior descending coronary (LAD) ligation. Mice were anesthetized with a mixture of 100 mg/kg ketamine and 10 mg/kg xylazine, intubated and ventilated with air. A left lateral thoracotomy was performed to expose the heart. Following pericardiotomy, the LAD was visualized and ligated with a 6–0 prolene suture with an atraumatic needle. Successful ligation was confirmed by bleaching and hypokinesis of the perfused myocardium. Following expansion of the lungs by compression of the exhalation tube, the thorax and the skin were closed with 6–0 prolene sutures. The mice were ventilated until spontaneously breathed, and all efforts were made to minimize suffering. Mice were kept separate on a warming plate until fully awake. The sham operation was performed similarly but without coronary artery ligation. Echocardiography parameters were assessed pre- and 24h or 7days post-MI. Animals surviving the 7-days ischemic period, and animals treated for 24 MI, were first evaluated for cardiac function by echocardiography and then sacrificed.

### Heart excision

Animals were placed in a chamber filled with anesthetic isoflurane vapor (5%) until deeply asleep. Hearts were then excised, rapidly rinsed in saline to remove excess blood, blotted dry and snap frozen in liquid nitrogen. Hearts excised after MI were immediately cut into infarct and peri-infarct tissues and then snap frozen. Tissue collection was accomplished within less than 1 min. Hearts were kept at -70°C until analyzed by Western blotting or designated to histological measurements, as we have previously described [30].

### Two-dimensional guided M-mode echocardiography

Animals were lightly anesthetized under continuous inhalation of 1.5% isoflurane. Two-dimensional (2D) guided M-mode echocardiography was performed using an echocardiogram (Siemens 512, Sequoia, PA, USA) equipped with a 15-MHz linear transducer at baseline and following LAD ligation. The 2D mode in the parasternal long-axis view was used to monitor the heart. From this view, an M mode cursor was positioned perpendicular to the interventricular septum and posterior wall of the left ventricle (LV) at the level of the papillary muscles. An M mode image was obtained at a sweep speed of 100 mm/s. Left-ventricular end-diastolic dimensions (LVEDD), and left-ventricular end-systolic chamber dimensions (LVESD) were evaluated. The percentage of left-ventricular fractional shortening (FS) was calculated as [(LVDD—LVESD) / LVEDD] ×100 [30].

### Drug treatments

6-month-old WT and αMUPA mice were used for all drug treatments. The indicated materials were injected daily for 3 days. LAD ligation was inflicted 30 min after the last injection. The AF489 neutralizing-leptin antibody [31] or a nonspecific goat IgG control (8μg each) was injected through the tail vein. For treatment with pharmacological drugs, WT and αMUPA mice were each randomly assigned to one of 4 groups and injected intra-peritoneally with the drug or the corresponding solvent vehicle. AG-490 (40 mg/kg body weight) and Wortmannin (2.5 mg/kg) were injected intra-peritoneally.

### Serum separation and analyses

Food was removed at 20:00h and mice were kept in a new cage for 12h before blood collection. Blood was collected through a submandibular vein (~600 μL). Blood samples were kept at 4°C to allow clotting and centrifuged at 3000 rpm for 5 min. Serum was collected and stored at -20°C. Serum levels were determined for adiponectin and leptin (R&D Systems Inc., Minneapolis, MN, USA) using ELISA kits, according to the manufacturer's instructions. Serum levels of CK were determined using a commercial Olympus OSR6126 kit (Center Valley, PA, USA) [30].

### Histopathology

To measure the area at risk, Evans Blue dye (1% in saline) was injected into the coronaries through retrograde perfusion after aorta cannulation as we have previously described [32], a procedure leaving the area at risk unstained. The midventricular heart region was cut and fixed in 4% formalin and embedded in paraffin. 3μm transverse sections were obtained from the LV anterior wall. To assess infarct size, 3mm-thick midventricular viable heart sections were put in a 1% solution of 2, 3, 5-triphenyl tetrazolium chloride (TTC) in a phosphate buffer for 10 min at 37°C. TTC stains the viable tissue red while the necrotic tissue remained discolored. Sections were fixed overnight in 4% formaldehyde to enhance the contrast between stained and unstained tissue. The sections were then placed between two cover slips and digitally photographed using a Nikon Coolpix 5000 camera (Nikon Imaging Japan Inc.), at a resolution of 1400×960 pixels, and quantified by using ImagePro PLUS software (Media Cybernetics, USA). The area of irreversible injury (TTC-negative) is presented as a percentage of LV area. To assess cell death, TdT mediated dUTP nick end labeling (TUNEL) staining was carried out on LV transverse sections using commercial kits (Roche Applied Science, South San Francisco, California, USA). Quantification was performed in 4–8 hearts on 5 sections from each mouse (5×fields each) and the numbers of healthy or apoptotic cardiomyocytes were counted. The percentage of apoptotic cells was considered the percentage of the total number of cells that were TUNEL-positive, using ImagePro PLUS software (Media Cybernetics, USA). To assess neutrophil infiltration, the following steps were undertaken for LV sections: treatment with rat anti-mouse neutrophil antibody MCA771GA (1:3000, AbD Serotec, UK); incubation with biotinylated donkey anti-rat (1:500, Jackson ImmunoResearch Laboratories, PA, USA), incubation with peroxidase-conjugated streptavidine (1:1,000, Jackson ImmunoResearch Laboratories, PA, USA); developement with 3, 3′-diaminobenzidine substrate. Quantification was performed on sections from 4–6 mice (5×fields each section) using ImagePro PLUS software (Media Cybernetics, USA) [30,32].

### Western blotting

Tissue samples excised from the peri-infarct area 24h post MI were homogenized in lysis buffer and quantified for protein levels using a commercial assay (Bio-Rad, CA, USA). Western blotting was performed according to standard procedures as we have previously described [30]. Protein samples (60μg) were applied to sodium dodecyl sulfate (SDS) polyacrylamide gel (10–15%), electrophoresed under denaturing conditions and electrotransferred onto nitrocellulose membranes (Bio-Rad) for 1h at 100V. Membranes were blocked with 3% BSA in Tris-buffered saline (TBS) for 1h at room temperature. Primary antibodies for phosphorylated AKT (Ser473), STAT3 (Tyr705) (1:1,000; Cell Signaling Technology, Beverly, MA) or IKB (nuclear factor of kappa light polypeptide gene enhancer in B-cells inhibitor, alpha) (1:1,000; Santa Cruz Biotechnology, Dallas, Texas) were used in TBST with 3% BSA overnight at 4°C. Immuno-detection of actin with mouse monoclonal anti-β actin (Santa Cruz Biotechnology, Dallas, Texas) was performed to serve as an internal control. Dye 600 or 800 secondary antibodies were added at a concentration of 1:15,000 for 1h at room temperature (LI-COR Biosciences, NE, USA). Detection was carried out with the LI COR Odyssey. Quantification of signals was carried out with the Odyssey program or Image J. The ratio between the intensity of the band of the tested protein and the intensity of the corresponding actin band was calculated for normalization/expression of results.

### Statistical analysis

Animals were assigned to groups randomly. All values are expressed as mean±SEM. When two mouse groups were compared, the unpaired t-test was conducted. When multiple time-point measurements were taken over time (the isolated heart), repeated-measures analysis was performed (one way ANOVA), followed by the Bonferroni test. Differences in mortality rates, by Genotype and Mouse Age, with Genotype and Age interaction, were assessed by Cox Proportional Hazards Survival model. Differences in mortality rates by age, within Genotypes, were compared by using the Kaplan-Meier survival test including p of the Wilcoxon test. *p<0.05 was considered statistically significant; **p<0.01. Statistical analysis was conducted using SAS Version 9.4.

## Results

### αMUPA mice show increased survival after MI

Ischemic tolerance is known to decline with age [2,3]. To determine whether age affects the αMUPA and WT heart differently in this respect, mice underwent LAD ligation *in vivo* for 7 days at the ages of 6 (young adults), 18 (aged) and 24 (senescent) months. Survival of WT and αMUPA mice was monitored throughout the ischemic period (Fig 1A and 1B). Both mouse genotype and age had a significant effect on survival (p<0.04 and p<0.03, respectively), with no interaction between the parameters (Cox Proportional Hazards Model). While the age effect was non-significant (p = 0.45) in αMUPA, it was significant (p<0.001) in WT mice (From Kaplan-Meier with Wilcoxon). At the youngest age, survival after the entire ischemic period was 50% and 63% in WT and αMUPA mice, respectively (p>0.05). At 18 months, the survival rate was 38% and 59%, respectively (p< 0.05). None of the senescent WT mice survived the first ischemic day while senescent αMUPA showed ~70% survival (p<0.005). 50% of the latter group survived the entire 7-day ischemic period. Overall, these results show that senescent αMUPA mice consistently demonstrated a youthful survival rate.

### αMUPA mice demonstrate reduced cardiac damage after MI

Echocardiography conducted in the mice surviving the entire ischemic period indicated significantly better LV functions, such as improved Left ventricular diastolic (LEVDD) and systolic (LVESD) diameters and increased Fractional shortening (FS), in αMUPA mice compared to the age-matched WT mice (Table 1), indicating a reduced age-dependent functional deterioration in the heart. It was also noted that the sham operated mice in both mouse genotypes did not display any decline in cardiac function at all ages and they survived the entire ischemic period (Table 1).

In our female mouse cohort, we also monitored several features previously reported to differ in αMUPA mice. Following survival throughout an 18 months period under the standard husbandry indicates ~30% increase (P<0.05) in αMUPA (results not shown), recapitulating the previously reported increased longevity in these mice [7,33]. Table 2 presents body weight (BW), total ventricular weight (VW) and food intake in WT and αMUPA mice. αMUPA mice weighed about 16% and 12% less than FVB/N mice at 6 and 18 months of age, respectively, had lighter heart weight and showed 17% and 13% reduction in daily food intake at the two ages, respectively.

**Table 2:** Body weight (BW), total ventricular weight (VW) and food intake in WT and αMUPA mice.

	6 months		18 months	
	WT	αMUPA	WT	αMUPA
BW (g) at baseline	26.0±0.45	21.5 ±0.3*	29.3±1	25.7 ±0.7*
VW (mg)	114.8±5.3	85.2±3.3**	114.4±1	98.8±7.7*
VW/BW (%)	4.4±0.2	4.0±0.2	3.9±0.1	3.9±0.1
Food (g)	3.74 ±0.07	3.08±0.08*	4.20±0.18	3.65±0.10*

Data are means ±S.E. (n = 5).

* P< 0.05, αMUPA vs. WT,

**P< 0.002, αMUPA vs. WT

**Fig 2:**
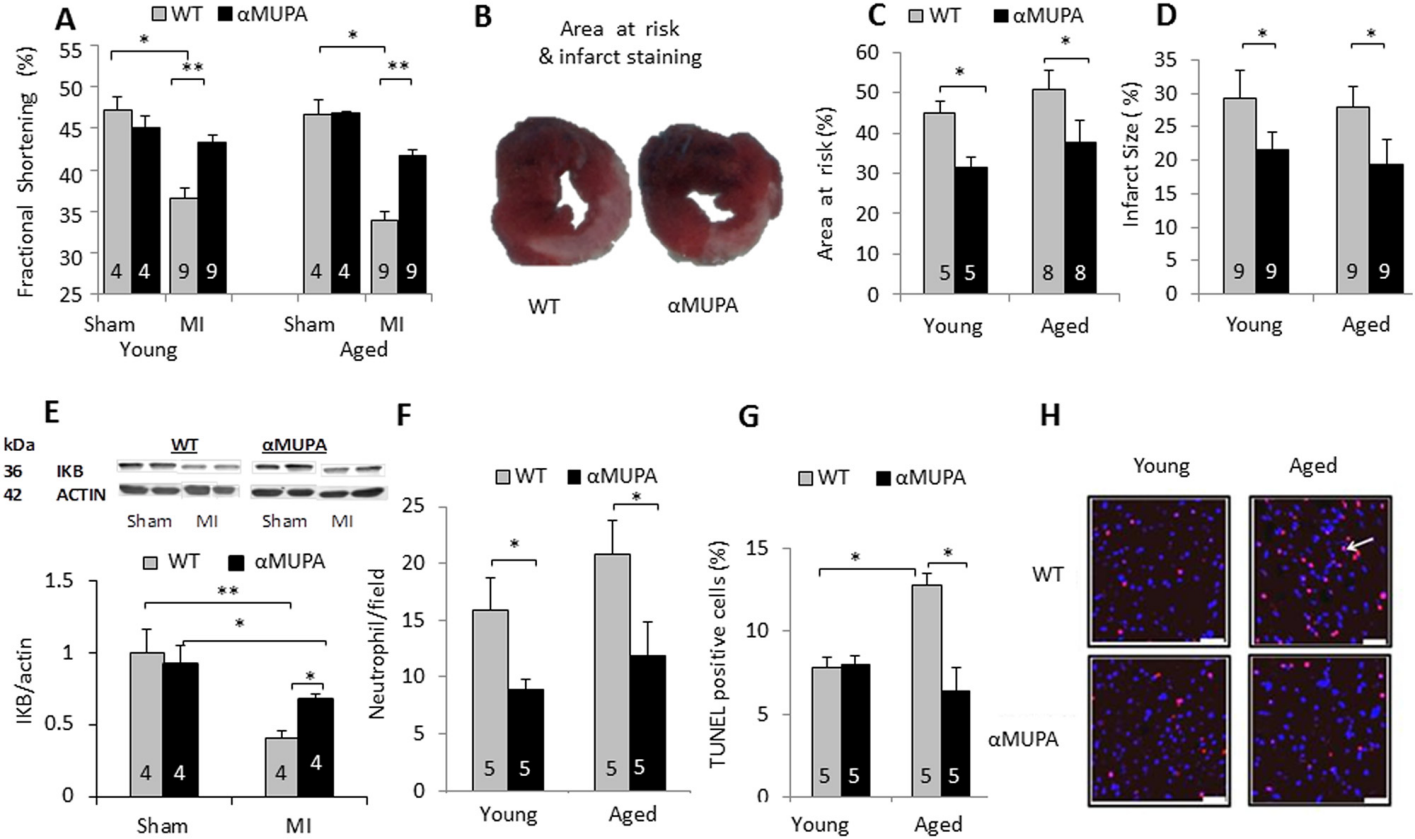
Functional and histological parameters after 24h MI in young and aged αMUPA and WT mice. 6- and 18-month-old mice (young and aged, respectively) were subjected to LAD ligation for 24h and examined for functional and histological parameters. A, fractional shortening as measured by echocardiography; B, representative LV slices stained to delineate area at risk and infarct zone; C, D, quantitation of area at risk, and infarct size staining, respectively. E, IKBα levels analyzed in Western blots; F, quantification of neutrophil infiltration; G, quantitation of TUNEL staining F, TUNEL staining for DNA fragmentation. Scale bar: 200 μm. The number of mice is indicated in the figure. *, P< 0.05; **, P< 0.01.

Next, we induced MI for 24h in young and aged αMUPA and WT mice. Cardiac function was tested by echocardiography and the LV was examined for the area at risk, infarct size, apoptosis or inflammation. The results show (Fig 2A) that FS values were similar (50.5%-51.8%) in sham operated mice irrespective of mouse type or age. However, after MI, FS was significantly higher in αMUPA (44.8±1.6% and 43.3±1.0%) compared to WT mice (37±1.2% and 34±1.0% in young and aged WT, respectively), indicating that young αMUPA mice can overcome more than half of the functional damage inflicted in the WT heart and, furthermore, senescent αMUPA mice maintain this capacity. The area at risk and infarct size were lower (P<0.05) in αMUPA mice at both ages (Fig 2B and 2D). Lower numbers of neutrophils (Fig 2F) and an increased level of IKBα (Fig 2E) were also observed in αMUPA mice, indicating a reduced inflammatory response. Also, similar numbers of cells stained for apoptosis after MI were detected in young WT and αMUPA mice. This number, however, doubled in the aged WT mice but did not change in aged αMUPA mice (Fig 2G and 2H). These results indicate that aged αMUPA mice maintain a youthful response. Interestingly, both numbers of infiltrating neutrophils and apoptotic cells showed differences between the genotypes in aged mice, whereas only neutrophil infiltration differed in young mice, suggesting that the inflammatory marker comprises a more sensitive marker for cardiac ischemic stress.

### αMUPA and CR mice demonstrate reduced cardiac damage after I/R

**Fig 3:**
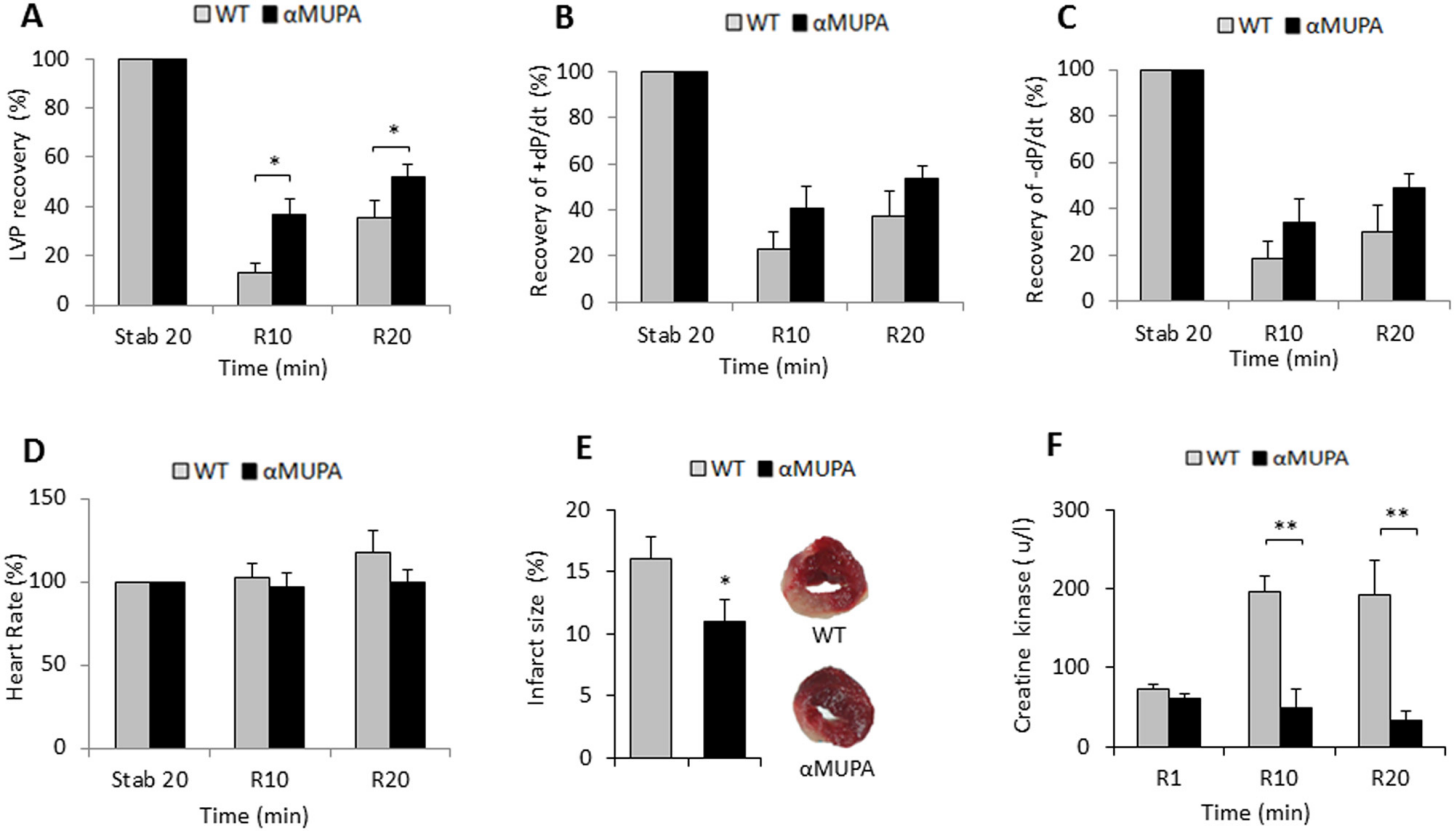
Functional and histological parameters after I/R in WT and αMUPA mice. Hearts excised from WT (n = 9) and αMUPA (n = 10) mice were treated for I/R as described in the Methods section. LVP was determined throughout the procedure. A, percentage of LVP recovery; B, maximum rate of LV contraction (+dP/dt max, mmHg/sec); C, maximum rate of LV relaxation (−dP/dt max, mmHg/sec); D, heart rate (beats/min); E, infarct size; F, creatine kinase released into the coronary effluent. LVP, +dP/dt max and -dP/dt max are presented as % of values measured in the stabilization period. Using ANOVA comparisons compared to WT hearts, αMUPA hearts demonstrated increased recovery of contractile parameters, (LVP, p<0.006) with a trend of higher values of positive dP/dtmax (p<0.13) and negative dP/dtmax (p<0.04) (Fig 3A–3C); a similar heart rate (Fig 3D), reduced infarct size (Fig 3E) and reduced levels of CK in effluent samples collected throughout the procedure (Fig 3F). Stab, stabilization; R, reperfusion. Data are means ±S.E.M. *, P< 0.05; **, P< 0.01

We compared αMUPA and WT mice for the response of the isolated heart to I/R. The results show that during the stabilization period all performance parameters (except for the heart rate) were higher in WT compared to αMUPA hearts (data not shown). Therefore, each heart served as its own control; i.e. we present the recovery throughout the subsequent reperfusion period as a percentage of the value measured in the stabilization period of the same heart. Compared to WT hearts, αMUPA hearts demonstrated increased recovery of contractile parameters, a similar heart rate, reduced infarct size (Fig 3E) and reduced levels of CK in effluent (Fig 3F). These results demonstrate reduced damage and a superior recovery rate in the αMUPA heart after I/R.

**Fig 4:**
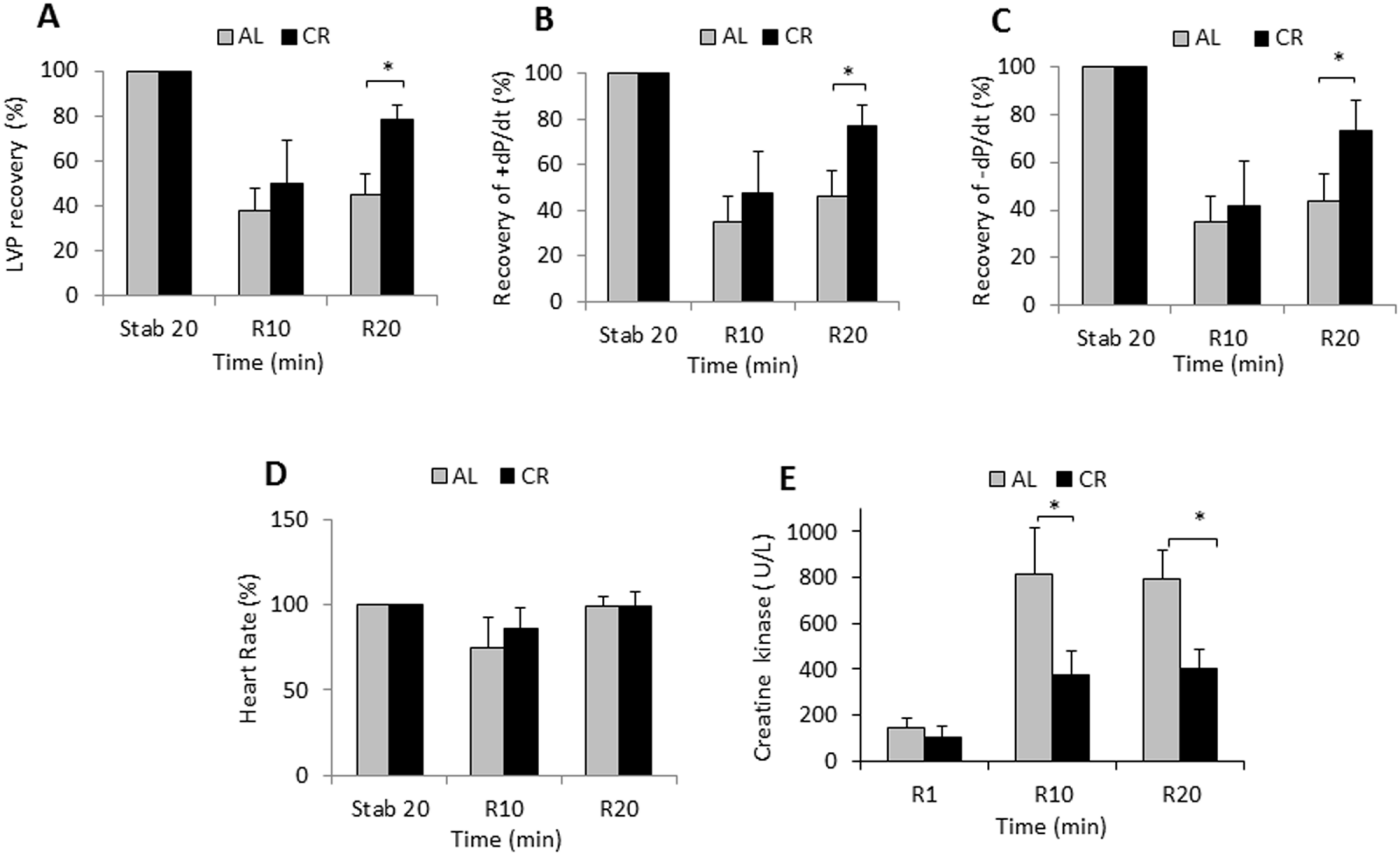
Functional and histological parameters after I/R in WT mice fed AL or CR. FVB/N mice were treated for short term CR (n = 4) or fed AL (n = 8) as described in the Methods section. Hearts were excised and treated for I/R as described in Fig 3. Parameters were monitored and are expressed as described in Fig 3. A, percentage of LVP recovery; B, maximum rate of LV contraction (+dP/dt max %); C, maximum rate of LV relaxation (−dP/dt max%); D, heart rate (beats/min); E, creatine kinase released to the coronary effluent. Stab, stabilization; R, reperfusion. Data are means ±S.E.M. *P< 0.05.

To evaluate whether αMUPA mice resemble CR mice in this behavior, we tested FVB/N, the ancestral αMUPA mice that are rarely used in CR and ischemic fields. We subjected young WT FVB/N mice to short-term CR and tested them in the I/R model compared to WT mice fed AL. The results show that post-ischemic recovery of contractile functions was considerably better in the CR group (Fig 4A–4C) and CK release was reduced (Fig 4E) while the heart rate was similar in the two groups (Fig 4D). Overall, the response of the CR heart generally resembled that of the αMUPA heart.

### αMUPA and CR mice differ in serum adipokines

**Fig 5:**
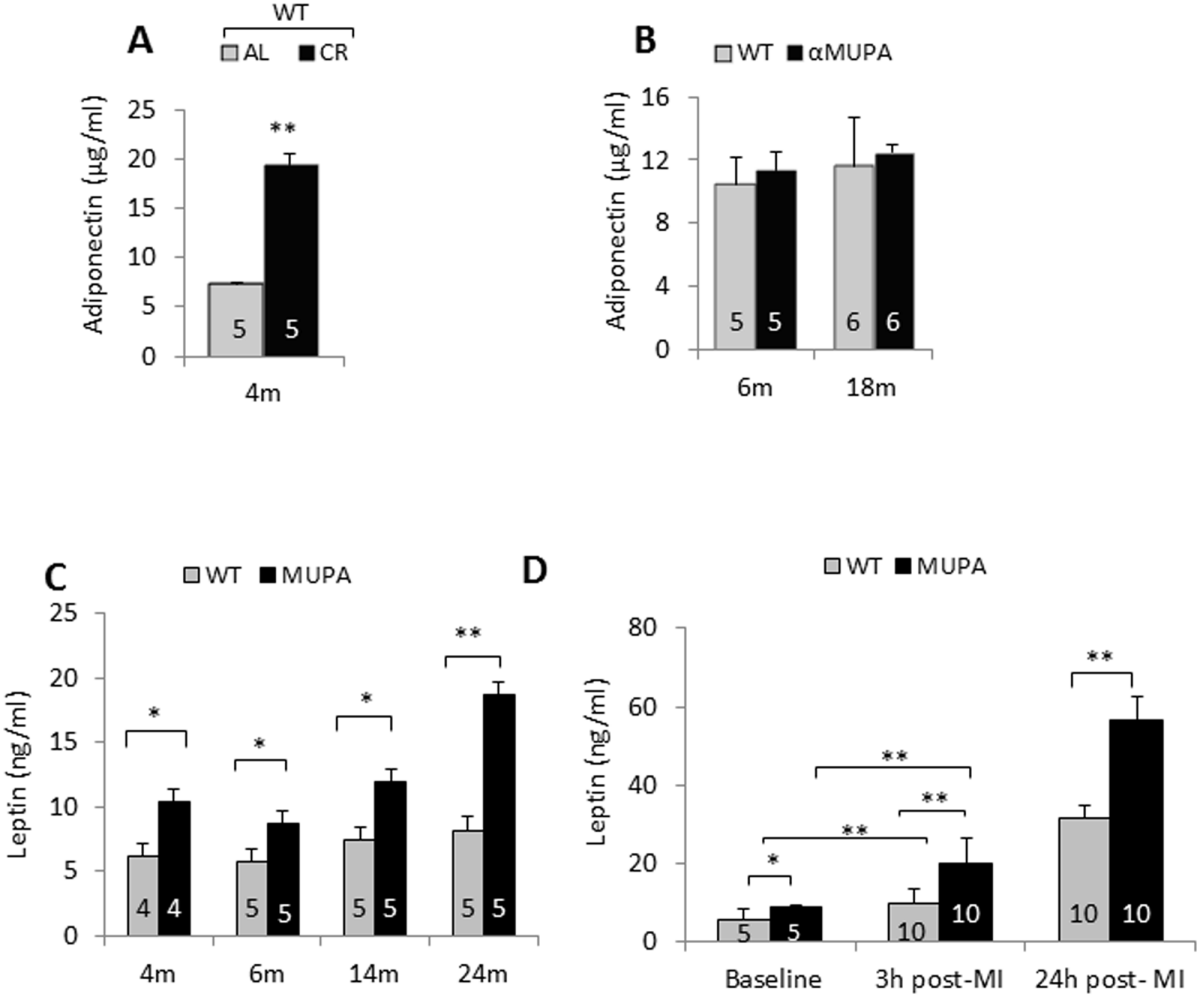
Levels of adiponectin or leptin in the serum of WT mice fed AL vs. CR, and αMUPA vs. WT mice fed AL. A, Serum samples collected at baseline from WT mice fed CR or AL were tested for adiponectin levels; B, Serum samples collected at baseline from WT and αMUPA mice were tested for adiponectin levels; C, As in B, but the test was for leptin; D, Serum samples collected from WT and αMUPA mice before, 3h and 24h after LAD ligation were tested for leptin levels. The number and age of mice are indicated in the figure. Data are means ±S.E.M. m = months. *p<0.05, **p<0.01

Previously, adiponectin has been shown to increase in the plasma after CR [16,17] and has been implicated in CR-induced cardioprotection [16]. Conversely, leptin was shown to decrease under CR or fasting [11,12]. We therefore compared the baseline levels of the two adipokines in αMUPA vs. WT mice and in WT (FVB/N) fed CR vs. *ad libitum* (AL). The results show that serum levels of adiponectin were significantly increased after CR compared with AL-fed mice (Fig 5A), but were similar in WT or αMUPA mice at the two ages tested (Fig 5B). In contrast, baseline leptin levels showed a life-long ~60% increase in αMUPA compared to WT mice (Fig 5C). Previously, circulating leptin levels were shown to increase after prolonged MI [34]. We therefore measured leptin levels in WT and αMUPA mice after LAD ligation. The results (Fig 5D) show a gradual elevation of leptin in both genotypes after 3h and 24h MI; however, the αMUPA levels significantly doubled (p<0.001).

### Leptin neutralizing antibodies, AG-490 and Wortmannin, abrogate cardioprotection in αMUPA mice

**Fig 6:**
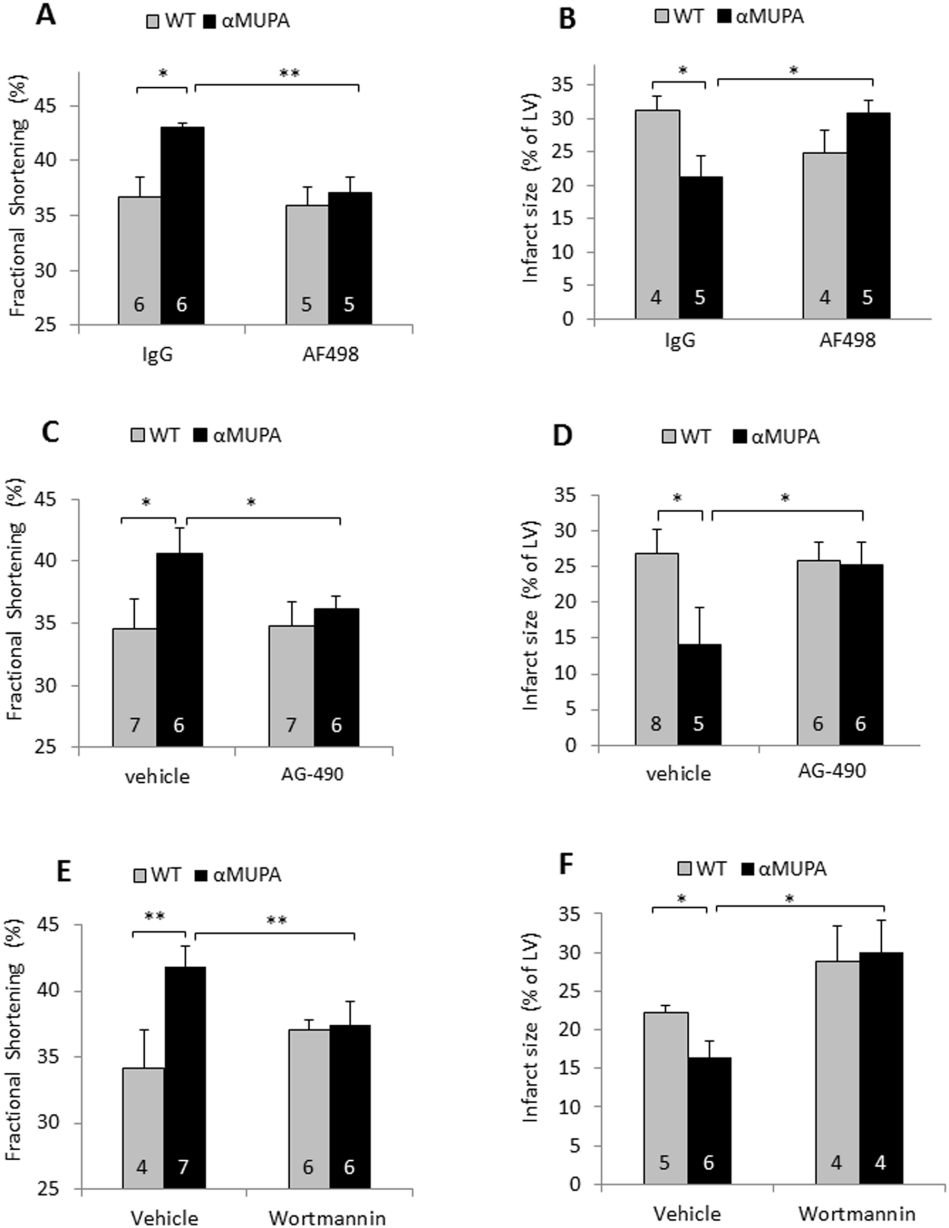
The effect of leptin neutralizing antibodies and inhibitors of leptin signaling on functional and histological parameters after 24h MI. A, B, 6-month-old αMUPA and WT mice received 3 daily injections with AF498 antibodies vs. nonspecific IgG; C, D, with AG-490 vs. vehicle; E, F, with Wortmannin vs. vehicle. 30 min after the last injection mice were LAD ligated for 24h. Fractional shortening was determined by echocardiography (A, C, E). Infarct size was evaluated by TTC staining (B, D, F). Data are means ±S.E.M. *, P< 0.05; **, P< 0.01. The number of mice is indicated in the figures.

As our results demonstrated no increase in αMUPA adiponectin, we tested the possibility of leptin being involved in αMUPA cardioprotection. To that end, we applied the leptin neutralizing antibody AF498 [31] and two known pharmacological inhibitors of leptin signaling, AG-490, a JAK2 inhibitor, and Wortmannin, a PI3K inhibitor. αMUPA and WT mice were pretreated with the aforementioned inhibitors or the corresponding vehicles and subjected to 24h MI. The results demonstrate (Fig 6A) that FS was significantly higher in αMUPA compared with WT mice (41–43% vs. 34–37%, respectively) in IgG- or vehicle-treated mice. After pretreatment with the leptin antibodies, αMUPA and WT mice showed similar levels of 35–37% FS (Fig 6A and 6B) indicating that leptin neutralization abrogated the superior αMUPA FS. The antibodies also abolished the infarct size reduction in αMUPA mice (Fig 6B). Pretreatment with AG-490 yielded results similar to those seen with the AF498 antibodies (Fig 6C and 6D); it affected only αMUPA mice, significantly increasing infarct size and reducing FS (from 42.3±2.6% to 36.2±2.4%, a value similar to that of the corresponding WT mice, 34.8 ±2.6%). Wortmannin exerted similar effects specifically in αMUPA mice (Fig 6E and 6F). Collectively, these results demonstrate that leptin; JAK2 and PI3K are involved in protecting the αMUPA heart against ischemic injury. Importantly, the leptin antibodies and both pharmacological inhibitors did not reduce the levels of αMUPA FS and infarct size below that of the WT values, indicating that they specifically inhibit the αMUPA beneficial increment without affecting any putative protective mechanism involving JAK2 and PI3K that could be shared by αMUPA and WT mice.

### The effect of leptin neutralizing antibodies, AG-490 and Wortmannin on signaling components in αMUPA and WT myocardia 24h post MI

**Fig 7:**
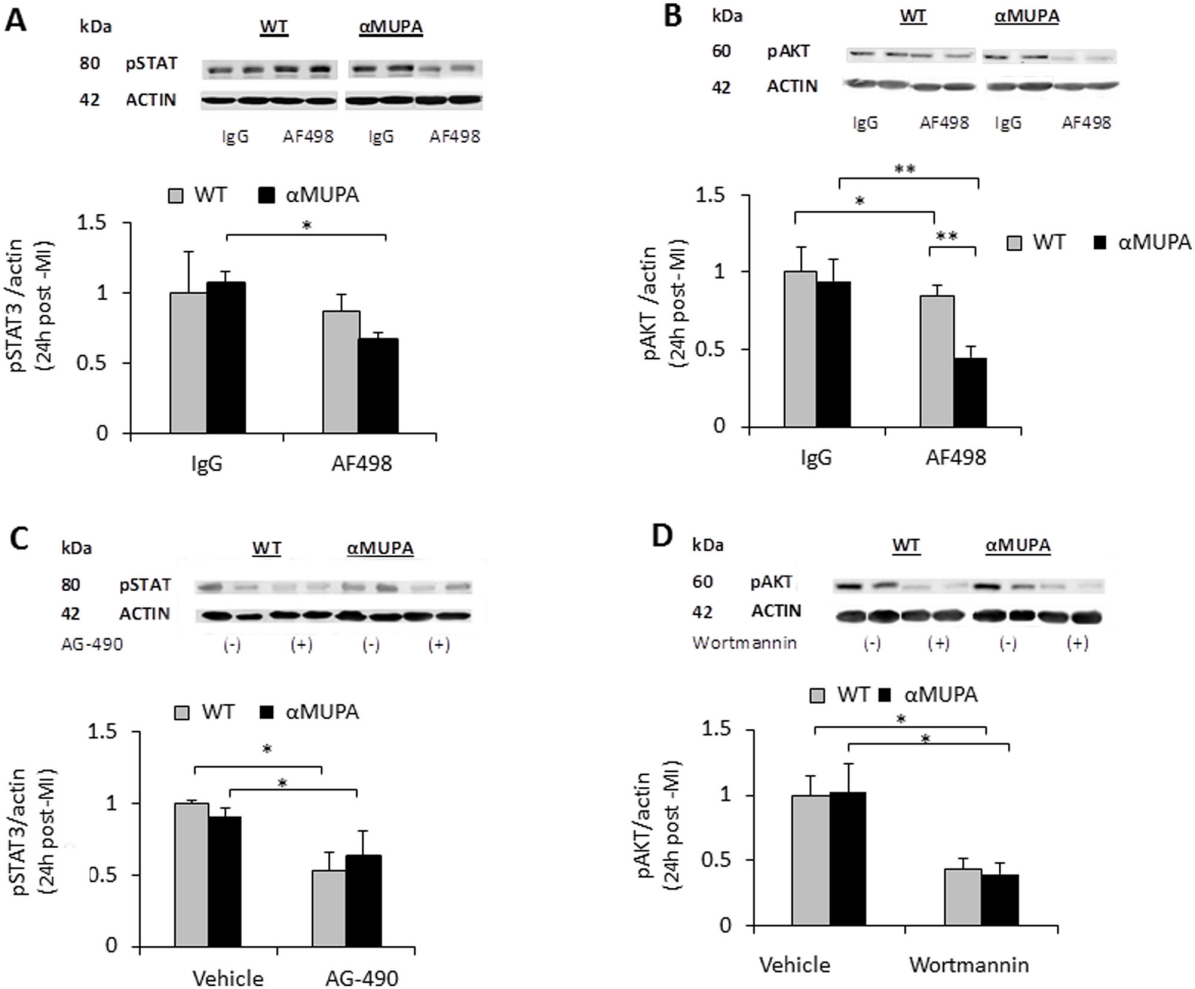
The effect of leptin neutralizing antibodies and inhibitors of leptin signaling on the levels of phosphorylated STAT3 and AKT after 24h MI. Hearts were excised from the mice described in Fig 6, that were treated with the indicated inhibitors or corresponding vehicles and subjected to LAD ligation for 24h. Samples extracted from the LV area were tested by Western blotting using antibodies for pAKT, pSTAT3 and actin. The ratio between the intensity of the band of the tested protein and the intensity of the corresponding actin band was calculated. The result obtained for the WT/IgG or WT/vehicle group was defined as one. Data are means ±S.E.M. *, P< 0.05; **, P< 0.01; n = 4–7 in each group.

We tested whether pretreatment with the leptin neutralizing antibodies, AG-490 or Wortmannin indeed exerted inhibition on the corresponding target molecules after 24h MI. At the end of the experiment described in Fig 6, hearts were collected and tested for the phosphorylation levels of STAT3 and AKT. In the presence of only IgG or vehicles, there were no differences between αMUPA and WT hearts in the levels of pSTAT3 and pAKT after MI (Fig 7A and 7B). The leptin neutralizing antibody AF498 significantly reduced the levels of pSTAT3 and pAKT specifically in αMUPA without affecting these molecules in the WT heart (Fig 7A and 7B). AG-490 reduced the levels of pSTAT3 in both mouse types (Fig 7C), and Wortmannin significantly reduced the levels of pAKT in both αMUPA and WT myocardia (Fig 7D). These results confirmed that the inhibitory capacity of all 3 inhibitors was sustained throughout the 24h ischemic period. The results also revealed that the pharmacological inhibitors did not discriminate between the mice and inhibited their target enzyme similarly in both genotypes. Yet, the inhibition of STAT3 or AKT phosphorylation correlated with reduced cardioprotection only in αMUPA mice, indicating no involvement of the two components in WT cardioprotection.

## Discussion

The present study demonstrates that long-lived αMUPA mice exhibit virtually a life-long increased tolerance to ischemic stress in the heart, indicating the retardation of cardiac aging. αMUPA cardioprotection is mediated through endogenously increased leptin levels and appears to involve the leptin/JAK2/STAT3 and leptin/JAK2/PI3K/AKT cascades.

### Cardiac aging is attenuated in αMUPA mice

Reduced ischemic tolerance is a hallmark of cardiac aging [2,3]. Therefore, our results indicate that cardiac aging is attenuated in αMUPA mice. This conclusion is clearly demonstrated firstly, by results showing that all senescent WT mice died within one ischemic day while half of the senescent αMUPA mice survived a 7-day ischemic period (Fig 1). Secondly, αMUPA mice surviving the ischemic period exhibited better LV functionality at each age (Table 1). The results also depicted that the number of apoptotic cells changed in aged WT mice while maintaining a youthful state in αMUPA mice, reinforcing the notion that aging is retarded in the αMUPA heart. Such a capacity has previously been reported for additional features, including circadian parameters [35,36], wound healing [37] and overall appearance [38]. It is likely that the lifelong increased ischemic tolerance is one longevity determinant in αMUPA mice. This study further establishes αMUPA as a mouse model by which to understand mechanisms leading to attenuated aging.

### Cardioprotection and adipokine levels in αMUPA and CR mice

**Fig 8:**
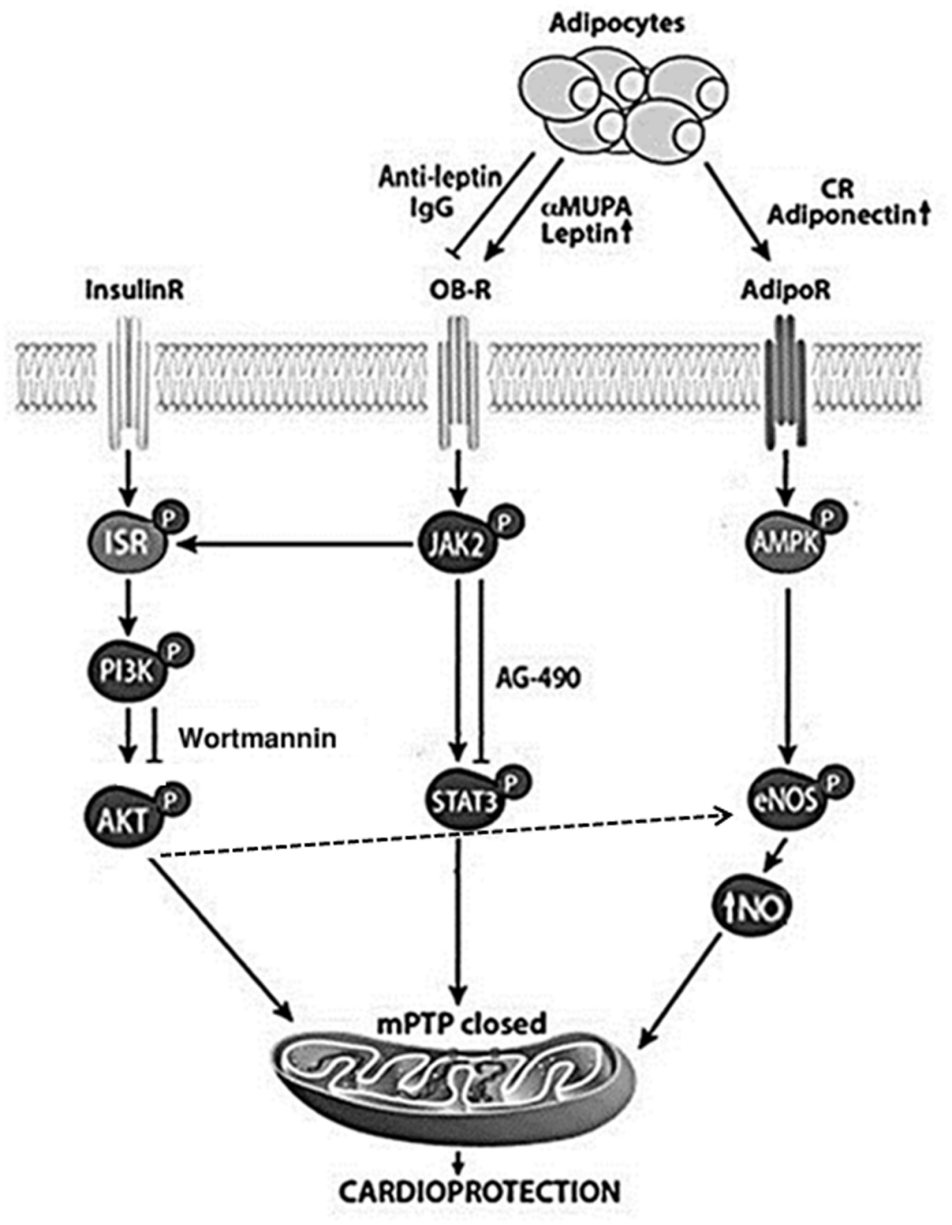
A scheme illustrating distinct cardioprotective pathways in αMUPA and CR mice. αMUPA mice show a life-long increase in leptin levels at baseline and twice elevated leptin levels post MI. Anti-leptin antibodies or inhibitors of leptin signaling through the leptin receptor OB-R (Wartmannin and AG-490) abrogated the αMUPA cardioprotection, suggesting the involvement of leptin, pSTAT3 and pAKT in the protective process. pSTAT3 and pAKT, both implicated in the inhibition of mPTP opening, could lead to cardioprotection by preserving the closed state of mPTP [42,43,44]. Conversely, CR leads to a decrease in leptin but an increase in adiponectin levels and AMPK activation, a pathway involved in CR-induced cardioprotection [16]. Phosphorylated AMPK, as well as pAKT, can in turn activate endothelial nitric oxide synthase (eNOS) and thus elevate the generation of nitric oxide (NO), another component implicated in the inhibition of mPTP opening [42].

Our results show that young and aged αMUPA mice exhibited a ~5% better FS after LAD ligation for 7 days or 24 h, indicating that they can overcome 40–50% of the functional damage detected in the WT heart (Table 1). This response was accompanied by a significant reduction in the area at risk, infarct size and neutrophil infiltration at least after 24h MI (Fig 2). The results for I/R experiments in isolated young αMUPA hearts also illustrated a significantly superior mechanical capacity along with reduced tissue damage (Fig 3). Collectively, these results indicate that the αMUPA heart is better protected against ischemic or I/R injury compared to the WT heart. Conversely, we did not detect differences in PR intervals (not shown) or heart rate (HR) between αMUPA and WT mice after ischemia at the three ages tested, indicating no differences in the cardiac electrical capacity between the two genotypes. The cardioprotection found in αMUPA mice after I/R was generally similar to that seen here after short-term CR in αMUPA ancestral FVB/N mice (Fig 4), a strain rarely used in CR experiments and reported to be relatively resistant to I/R injury [39]. Our results for CR mice are supported by literature data in showing cardioprotection after I/R [16,25] and increase of adiponectin [16,17], an adipokine previously implicated in CR-induced cardioprotection after I/R [16]. In contrast, no change was detected in αMUPA adiponectin. Collectively, these data suggest that ischemic tolerance after I/R is mediated in αMUPA and CR mice through distinct pathways rather than merely via the reduced energy intake common to the two cases (illustrated in Fig 8). We assume that, similarly to the baseline increase in adiponectin under CR, the baseline increase of leptin in αMUPA mice could have an impact in the isolated heart under I/R, leading to damage reduction.

### Leptin plays a causative role in αMUPA cardioprotection after MI

Leptin is a 16 Kd adipokine of a pleiotropic nature, the plasma levels of which are proportional to the body fat content [10,11,12,13]. αMUPA mice appear to be an exception as they display increased leptin levels along with reduced fat mass [8], resembling, in this sense, the inverse relation between adiponectin levels and fat mass under CR. The reason for this distinctiveness in αMUPA mice is not yet clear. Leptin however is responsible for the lean phenotype in αMUPA mice [9]. Leptin is the primary hormone through which the hypothalamus and possibly the brain stem sense the nutritional state and modulate food intake and energy balance [11,40]. In the heart, leptin controls several metabolic activities including fatty acid oxidation, fatty acid delivery and anti-lipotoxicity, as well as cardiac sympathetic nerve activity [10,11,40]. Leptin exerts its multiple effects via signaling pathways elicited through the leptin surface receptor (ObR) which is distributed in brain and peripheral tissues including the heart [40,41], thus leading to central and local effects. The binding of leptin to ObR recruits JAK2, a tyrosine kinase that in turn phosphorylates several signaling molecules including STAT3, a transcription factor acting in metabolic regulation, and the Insulin Receptor Substrate (IRS), a key molecule in the insulin pathway. Phosphorylated IRS activates the PI3K/AKT cascade [40,41] implicated in the Reperfusion Ischemia Salvage Kinase (RISK) pathway in the myocardium [42].

Our results confirm that inhibiting leptin or two steps in leptin signaling prior and throughout the ischemic period abrogated the post-ischemic αMUPA advantage in the heart. Western blot analysis of myocardial tissues indicates that all three inhibitors used were still effective at the end of the 24h ischemic period. The anti-leptin antibodies reduced the levels of pSTAT3 and pAKT primarily in the αMUPA myocardium, suggesting that these two components are virtually leptin-independent in the WT heart. Both AG-490 and Wortmannin appear to inhibit their target enzyme in the heart without discriminating between WT and αMUPA mice; yet only in the latter mice this inhibition increased cardiac injury after MI, suggesting no involvement of pSTAT3 and pAKT in WT cardioprotection. Overall, these results suggest that WT and αMUPA mice differ in their intrinsic cardioprotective mechanisms, which are leptin-dependent in αMUPA and leptin-independent in WT mice. It is not yet clear why the post-MI rise in leptin does not contribute to cardioprotection in the WT heart and whether the FVB/N genetic background and/or the unique αMUPA metabolic state contribute to this difference between the two genotypes. Collectively, however, these results indicate that the increased circulating leptin protects the αMUPA heart against ischemic damage by triggering signaling locally in the myocardium. pSTAT3 and pAKT appear to be both obligatory components downstream in the leptin protective pathway. The two components were previously shown to mitigate the opening of the mitochondrial permeability transition pores (mPTP), a key event leading to mitochondrial dysfunction and cardiomyocyte death [42,43]. We hypothesize that pSTAT3 and pAKT could cooperate at that step to preserve cardiomycyte survival (Fig 8). Further investigation is needed to resolve the leptin-induced protective pathway in the αMUPA heart.

The capacity of leptin to induce cardioprotection through direct action in the heart has been recently demonstrated [44,45]. Exogenously added at reperfusion, leptin protected the mouse myocardium against I/R injury in a manner that could be linked to mPTP [46,47]. Administration of leptin, but not CR, reversed age-dependent cardiac hypertrophy in leptin-deficient ob/ob fat mice [48]. Studies with C57BL and leptin-deficient ob/ob mice have indicated that leptin signaling reduces the severity of cardiac dysfunction and remodeling and attenuates cardiac apoptosis after chronic ischemic injury [34,49]. Specific deletion of the cardiac ObR exacerbated ischemic heart failure and glucose metabolism along with a loss of leptin signaling [45]. Genetic depletion of cardiomycyte STAT3 abolished ischemic pre- and post-conditioning [50,51]. Prolonged MI or I/R increased circulating leptin and expression of leptin and ObR in the heart and visceral adipose tissue [34, 52] and enhanced leptin signaling in the myocardium.

Although leptin can regulate baseline physiology and metabolism in the heart through local and central effects [11,40], it is yet unclear whether leptin contributes to metabolic processes leading to the attenuation of cardiac aging in αMUPA mice. Also, as leptin can promote angiogenesis [53,54], and the αMUPA myocardium showed decreased area at risk 24h post-ischemia, we cannot exclude angiogenesis as one leptin-induced cardioprotective benefit. The slow kinetics of angiogenesis [53] however suggests that this process could be more effective later in recovery. We also wonder whether the beneficial circadian behavior previously detected in these mice [33,35,36] could be one determinant of the delayed aging, as the circadian clock is thought to contribute to cardiomyocyte metabolism [55]. Also, it is not apparent why αMUPA mice evade the well-known phenomenon of leptin resistance in contrast, for example, to transgenic mice overexpressing leptin [56]. αMUPA hyperleptinemia could be insufficiently high to lead to leptin resistance, or the sustainable leptin sensitivity could be related to the unique αMUPA metabolic state that, for example, combines high leptin, low fat mass and obesity-resistance [8].

In summary, αMUPA mice exhibit reduced functional and histological injury after MI or I/R along with the attenuation of cardiac aging. The latter trait is indicated by lifelong increased tolerance to ischemic stress. αMUPA mice show chronically elevated levels of leptin in the serum at baseline and enhanced increase of leptin under ischemia. Inhibitors of leptin and leptin signaling abrogated the post-MI cardioprotection in αMUPA mice, indicating a causative role in the protective process for endogenous leptin. These findings support the rationale for testing leptin in short-term therapy for the ischemic heart.
